# The impact of COVID-19 on medical students

**DOI:** 10.3205/zma001665

**Published:** 2024-02-15

**Authors:** Wenwen Wang, Genpeng Li, Jianyong Lei

**Affiliations:** 1 Division of Thyroid Surgery, Department of General Surgery, West China Hospital, Sichuan University, Chengdu, China

**Keywords:** COVID-19, medical students, medical education, postepidemic era

## Abstract

**Objective:**

The outbreak of COVID-19 has disrupted social order and placed a heavy burden on the healthcare system. The pandemic also has an unprecedented impact on medical students.

**Methods:**

We searched PubMed for articles related to COVID-19 and medical students from January 2020 to December 2022. A total of 5358 studies were retrieved and after screening, 176 studies were finally included in this review.

**Results:**

The impact of COVID-19 on medical students is widespread and profound. First reflected in the transformation of educational models. In the early days, education model quickly shifted from offline to online. In terms of clinical exposure, most students have been suspended from internships, while in some areas with staff shortages they have the opportunity to continue clinical work. Scientific research of medical students is also difficult to carry out due to COVID-19. The epidemic has also seriously damaged students’ mental health, and this impact won’t simply disappear with the improvement of the epidemic situation. The career intentions of medical students may also become firmer or change due to COVID-19. International medical electives have also been negatively affected by COVID-19 due to travel restriction. Even in the postpandemic era, with the gradual resumption of work, production and school, medical students are still affected in some ways by COVID-19.

**Conclusion:**

The COVID-19 pandemic has had a profound impact on both the education of medical students and their personal development. Through COVID-19, we should reflect on what models of medical education should be developed in the future. Based on the experiences learned from COVID-19, we believe that a more flexible blended education model may be the most promising.

## Zusammenfassung

**Zielsetzung:**

Der Ausbruch von COVID-19 störte die gesellschaftliche Ordnung und belastete das Gesundheitssystem immens. Die Pandemie hat zudem beispiellose Auswirkungen auf Medizinstudierende.

**Methoden:**

Wir durchsuchten PubMed nach Artikeln zu COVID-19 und Medizinstudierenden aus dem Zeitraum von Januar 2020 bis Dezember 2022. Wir fanden insgesamt 5.358 Studien, wovon nach der Auswertung 176 schließlich in diesen Überblick eingeschlossen wurden.

**Ergebnisse:**

Der Einfluss von COVID-19 auf Medizinstudierende ist weitreichend und tiefgreifend. Er wurde zunächst durch die Umwandlung der Ausbildungsmodelle deutlich: In der Frühphase wurde kurzfristig von Offline- auf Online-Unterricht umgestellt. Die Praktika im Bereich der klinischen Ausbildung wurden in den meisten Fällen unterbrochen. In einigen Gebieten, in denen Personalmangel herrschte, hatten Studierende jedoch die Gelegenheit, ihre klinische Tätigkeit fortzusetzen. Zudem ist die wissenschaftliche Forschung der Medizinstudierenden durch COVID-19 erschwert. Darüber hinaus wurde die psychische Gesundheit der Studierenden erheblich beeinträchtigt und diese Auswirkungen verschwinden nicht einfach mit der Verbesserung der epidemischen Situation. Die Berufsabsichten von Medizinstudierenden könnten durch COVID-19 verstärkt oder verändert werden. Auch internationale medizinische Wahlfächer werden durch COVID-19 aufgrund der Reisebeschränkungen negativ beeinflusst. Sogar in der Zeit nach der Pandemie mit allmählicher Wiederaufnahme von Arbeit, Produktion und Bildung werden Medizinstudierende in gewisser Hinsicht noch immer durch COVID-19 beeinträchtigt.

**Schlussfolgerung:**

Die COVID-19-Pandemie hat sowohl die Ausbildung als auch die persönliche Entwicklung von Medizinstudierenden erheblich beeinträchtigt. Sie zeigt, dass wir darüber nachdenken müssen, welche Modelle medizinischer Ausbildung künftig entwickelt werden müssen. Auf Grundlage der durch COVID-19 gewonnenen Erfahrungen sind wir der Meinung, dass ein flexibleres Blended-Learning-Modell am vielversprechendsten sein könnte.

## 1. Introduction

Since COVID-19 first emerged in China, it has profoundly impacted all aspects of society, including all levels of the education system [1]. The influence of COVID-19 on medical education is unprecedented due to the special nature of medical education, which is based on clinical practice [2]. As the primary constituents of the medical education system, students are the primary bearers of the impact of COVID-19 on medical education.

The epidemic undermines the traditional face-to-face teaching-learning model and presents unprecedented challenges to this learning approach [3]. In many countries, COVID-19 is already rapidly causing a radical restructuring of medical education; for example, education mode shifted from in-person to online approach [4]. Additionally, medical students were removed from the clinic [5] or allowed to graduate early prematurely and provisionally register to enter residency for patient care in some areas where there was a shortage of medical staff in the early stages of the outbreak [6]. These factors will affect their clinical exposure. Furthermore, exchange studies for medical students, such as international electives and clinical and scientific conferences, were also negatively affected by travel restrictions during the outbreak [7]. Apart from the learning aspect, due to a variety of factors, the epidemic has also damaged the mental health of medical students and may change their career intention. In short, the epidemic broke the peaceful study and life of medical students and affected them in every way.

At present, the global epidemic is gradually stabilizing, and the prevention and control of the epidemic is being gradually liberalized in an orderly manner. Many areas have returned to work and life, and students have returned to university. However, due to the constant mutation of the virus, the unfair distribution of vaccines and the relaxation of epidemic control measures in some countries, the global epidemic situation is still not optimistic. Medical students’ education and mental health are still affected in the postepidemic era. To gain a comprehensive picture of the effects of the disaster on medical students and draw experiences and lessons from it, we summarize the impact of the early and late stages of the epidemic on medical students’ learning, assessment, clinical exposure, mental health, career intentions, scientific research, and exchange studies.

## 2. Methods

**Figure 1:**
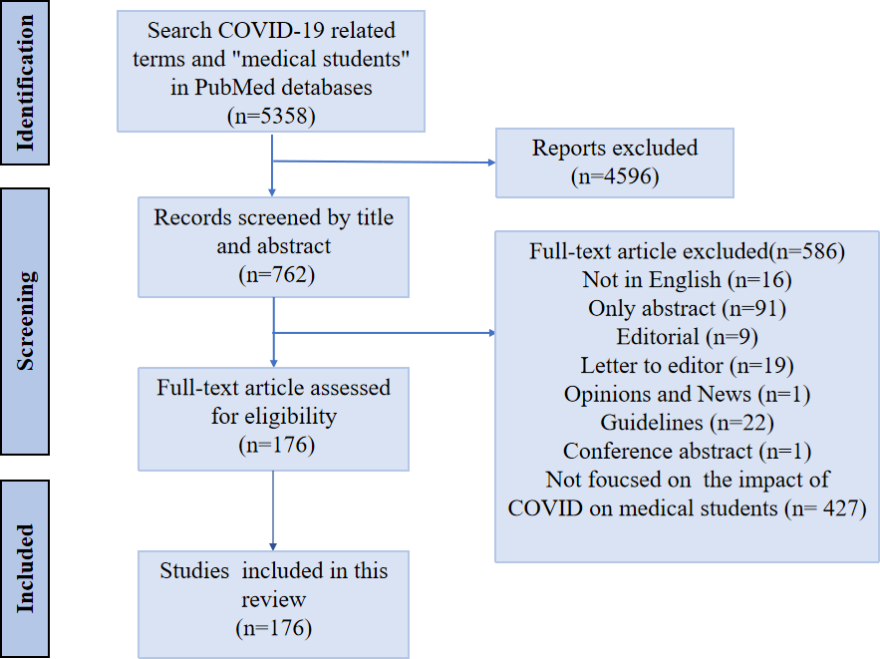
The flowchart of literature screening for this review

We searched for articles related to COVID-19 and medical students from January 2020 to December 2022 included in the PubMed database. The following search terms were used, “COVID-19”, “SARS-CoV-2”, “corona pandemic” and “medical students”. A total of 5358 articles were retrieved. After screening through the literature manager and manually, 176 studies were finally included. The detailed screening process is shown in figure 1 (Fig. 1). 

## 3. Online learning

### 3.1. Online learning

COVID-19 is a highly contagious and respiratory transmitted virus that is easily spread among the population [8]. To maintain social distance, many countries have closed educational institutions,and rapidly shifted in-person instruction to online learning [4]. Online learning is web-based learning that includes all measures of learning using the Internet and is increasingly common in medical education today, overcoming the barriers of time and space and allowing students to learn outside of the traditional classroom [9]. By 2025, online education is expected to become mainstream [10]. The COVID-19 pandemic may accelerate this trend [11]. Academic conferences are another way for medical students to learn new things. During the COVID-19 pandemic, many medical conferences and scientific meetings have been canceled or continued as webinars [12]. Online learning mode has played an important role in continuing medical education during the COVID-19 surge.

### 3.2. Strengths and weaknesses

The strengths and weaknesses in online learning mode are summarized in attachment 1 . Chinelatto et al. [13] reported that online learning may allow students to gain more free time as commute times are reduced and some extracurricular activities are cut. Andersen et al. [14] reported that increased time studying at home, and more flexible modes of study were perceived by students as advantages of online learning. Kaurani et al. [15] reported that online learning can provide students with more learning resources and facilitate the transformation of the learning process from passive teacher-led learning to active student-centered learning. Kaur et al. [16] reported that online learning can also offer students the potential to stay connected to their studies and continue discussing projects with faculty members while on lockdown. Moreover, being able to record, spending less on living expenses, being more convenient, and helping to improve students’ self-discipline are all seen as advantages of online learning [17], [18], [19]. These advantages of online learning may be an important reason for its widespread adoption by educational institutions as the primary learning method for students during the epidemic.

However, for medical students, online learning is a double-edged sword. The increased time studying at home also posed a problem of family disruption, such as noise, family responsibilities and obligations [20]. Additionally, while staying at home is convenient and comfortable, it also leads to the absence of face-to-face interaction [14]. Moreover, when the Internet provides substantial learning resource, the large flow of learning resources may lead to a surge in choice, which in turn may raise the rate of student burnout [21]. The disadvantages of online learning also include: lack of feedback from instructors [22], students are more likely to be bored and distracted [23], more equipment is required and there is a lack of hands-on practice [24], and prolonged exposure to screens can lead to visual dysfunction [25]. In some low- and middle-income areas, a lack of learning equipment and space, unstable Internet connections, are the main problems with online education [26]. For international students, the form of online learning will also make them fail to experience the campus atmosphere of study abroad and jet lag can cause chaos in their daily life [27]. 

The shortcomings of online education may be an important reason why it was not able to completely replace traditional face-to-face teaching in medical education.

## 4. Online exams

### 4.1. Online examination

The shift in online medical education has also witnessed a transition in examination methodology. During the epidemic, some medical schools used open-book exams (OBEs), multiple-choice, written summaries and other forms of assessment through online platforms to compensate for the lack of traditional exams [28]. Most clinical skill operations, except physical examination, can also be effectively evaluated by online objective structured clinical examination [29]. Online exams, similar to online learning, are also significant for continuing medical education during the epidemic.

### 4.2. Strengths and weaknesses

The strengths and weaknesses of online exams are summarized in attachmnent 2 . Perhaps the most intuitive advantage of online exams is to prevent delayed graduation and ensure continuity of medical education during the pandemic [30]. Additionally, OBEs can cultivate students’ critical thinking and improve their ability to analyze and solve problems in practical clinical work. It can also help students become self-directed learners and then keep pace with the development of medicine [31]. Moreover, because of the convenience and flexibility of online assessment, educators can keep abreast of students’ learning by scheduling more frequent tests [32]. Thus, more frequent testing may help reduce students’ anxiety [32]. 

**Figure 2:**
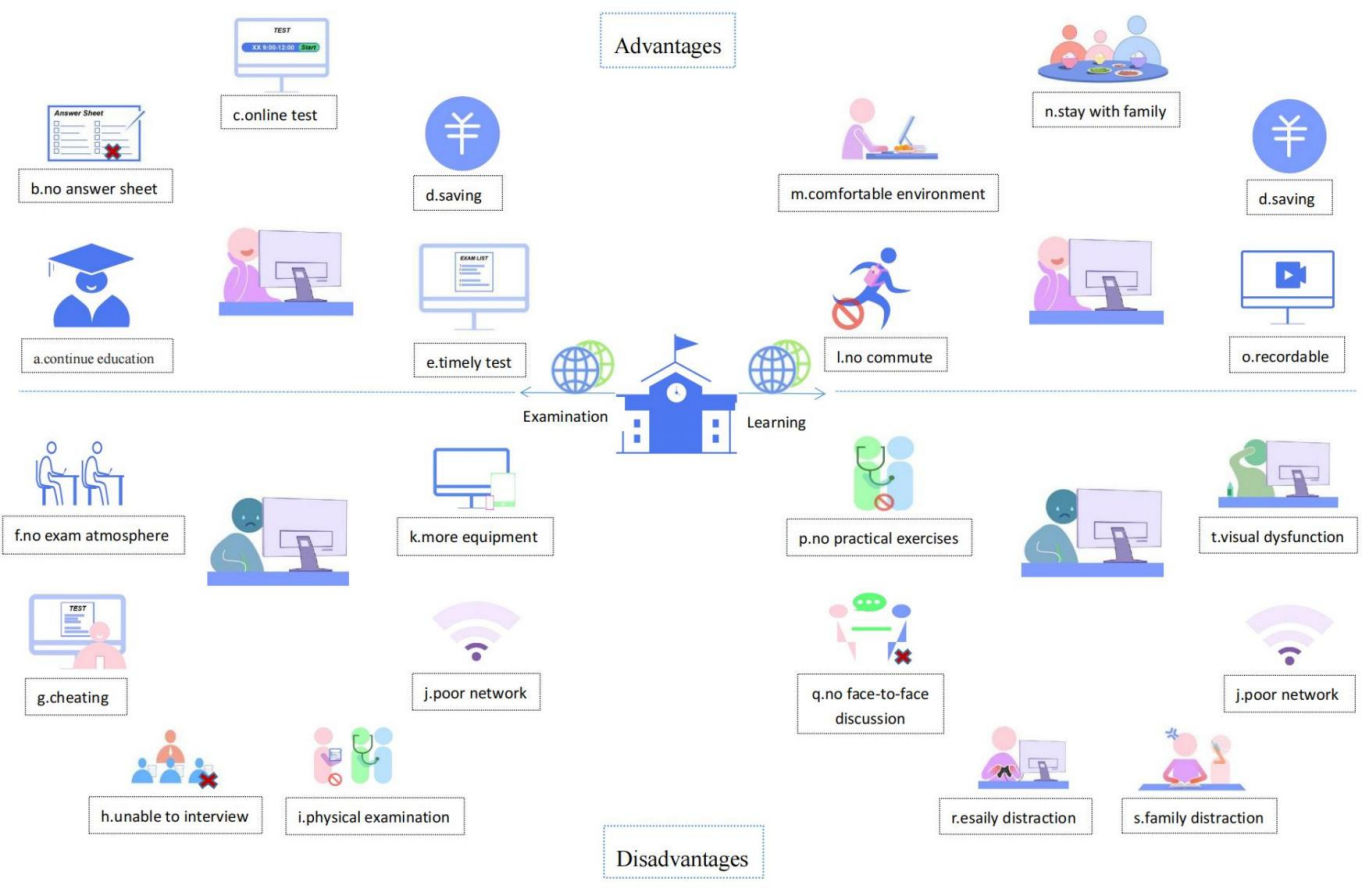
Advantages and disadvantages of online education a. maintain educational continuity; b. needn’t paint the answer sheet; c. convenient; d. saving; e. timely test; f. no exam atmosphere; g. cheating; h. no face-to-face interaction; i. lack physical examination; j. poor network connection; k. need more equipment; l. no commute; m. comfortable environment; n. stay with family; o. recordable; p. unsuitable for practical courses; q. no face-to-face discussion; r. easily distract; s. family distraction; t. visual dysfunction

However, Jaap et al. [33] found that more than 50% of students said online exams would make them more anxious, mainly because of concerns about network connection problems and a lack of an exam atmosphere. Poor internet connection can also negatively affect test scores [34]. Test cheating is a major problem in online exams [35]. Full electronic monitoring [36] and disrupting the order of the questions [36] helps to solve this problem. In addition, examiners may miss the opportunity to observe candidates through nonverbal communication due to the lack of face-to-face interaction in the online format [37]. The disadvantages of online exams may lead to inaccurate assessment (see figure 2 (Fig. 2)).

## 5. Clinical exposure

### 5.1. Negative impacts

Clinical exposure plays an integral role in the transition of medical students from students to doctors [38]. However, many medical students were removed from clinical rotations because they are potential carriers for COVID-19 and could become infected during training [39]. Also, their opportunities to participate in bedside teaching are also reduced [40]. Anwar et al. [41] note that COVID-19 has the most significant impact on clinical exposure in practice disciplines. The main reason was the decrease in elective surgeries due to COVID-19 [42]. 

Reduced clinical exposure for medical students means fewer opportunities to learn in real clinical settings. This is not conducive to their future transition to the role of a doctor.

### 5.2. Positive impacts

In some areas where there is a shortage of medical staff, medical students may be recruited to frontline work, which may have a positive effect on their clinical exposure. For example, during the peak of the pandemic, the Norfolk and Norwich University Hospital met the increased demand for the workforce by recruiting student clinical assistants to engage students in clinical work [43]. Aalborg University Hospital in Denmark has made a similar move [44]. Besides, students are encouraged to participate in primary care activities and public health programs such as community volunteering, vaccination campaigns, and telemedicine counseling service [38]. Participating in the fight against the pandemic may be the most unique experience of a medical student’s career and the wonderful learning opportunity that COVID-19 has provided them.

## 6. Medical education in the postepidemic era

COVID-19 has posed a great challenge to current medical education. However, it has also made us realize that in the postepidemic era, we need a better education system that is capable of dealing with all kinds of emergencies [2]. What kind of medical education model should we develop? Gadi et al. [45] reported that approximately 50% of medical students considered that online courses should be part of the regular curriculum. Zheng et al. [46] also found that 80% of students supported the continuation of some online instruction in the postepidemic era. However, because online education does not meet the practical curriculum, lacks clinical exposure and has other drawbacks, it cannot fully replace traditional face-to-face education in the postepidemic period [28]. Therefore, the blended education model combining online education and face-to-face education may be the future development direction of medical education [47]. In Tayem et al. [48] survey, 73.3% of medical students said they preferred blended teaching in the postepidemic era, where they learned theoretical knowledge through online courses and practical components through in-person learning. 

The current blended education model is immature. In the future development of the blended education model, on the one hand, it is necessary to make full use of technology to improve the online education model, and on the other hand, it is necessary for educators to design a more efficient way to combine online and offline education.

## 7. Mental health

### 7.1. Changes in mental health

COVID-19 also damages the mental health of medical students. In fact, information on both past epidemics (such as SARS, Ebola, etc.) and present COVID-19 suggested that many mental health problems, such as emotional distress, anxiety, depression, insomnia, suicidal ideation, etc., accompany the outbreaks [49]. Among all mental health symptoms, depression and anxiety are the major causes of mental burden worldwide [50]. Many studies [51], [52], [53], [54], [55] have reported that international students may face more serious mental health problems than domestic students during COVID-19.

### 7.2. Factors affecting mental health

The factors that impact student mental health are summarized in attachment 3 . Negative factors affecting students’ mental health are mainly as follows: extended daily screen time [56]; reduced clinical exposure and increased uncertainty about future educational and career prospects [57]; increased study workload and academic pressure [58]; social stressors, such as economic instability and insufficient food supply [59]; lack of physical exercise and recreational activities [59]; poor sleep quality, which is positively associated with the presence of health and psychological disorders [60]; pathological Internet usage [61]; quarantine or lockdown lead to the reduction of interpersonal communication and individuals with low perceived social support [62]. Smoking, alcohol consumption, previous poor physical health, history of mental illness, lack of exercise, low resilience, and COVID-19-like symptoms are also risk factors for mental well-being [63]. Visa restrictions, discrimination, sociocultural differences, jet lag causing late nights for online study, language barriers, years of stay in the host country and housing issues due to school closures are additional risk factors for international students that affect mental health [51], [52], [54], [55], [64] (see attachment 4 ). The impact of gender on anxiety during the pandemic is controversial [62], [65], [66], [67]. 

There are also many factors that contribute to mental health. We know that the Internet is flooded with information about COVID-19 during the epidemic, and it is good for our mental health to be properly informed about the right information [68]. In addition, research has demonstrated that a sense of control and stress training in daily life are both protective factors for mental health [69]. Wu et al. [70] revealed that a healthy diet, positive coping, and completion of vaccinations were protective factors affecting mental health. Moreover, residing in the city, with a stable family economic level, living with parents [62], having social support and emotional resilience, resilience training, outgoing and optimistic character, exercise and fitness [71], trust in government [72] and the health care system [73] all play a positive role in preventing negative emotions. Understanding the factors that positively affect mental health helps us take steps to address mental health issues that arise during an epidemic and to improve mental health.

### 7.3. Mental health in the postepidemic era

In the postepidemic era, the overall situation of the epidemic is gradually improving. However, does that mean the negative effects of the pandemic on mental health will fade as the pandemic improves? Wu et al. [70] also found comparable rates of anxiety symptoms and depressive symptoms (27.54% vs. 27.58%) and lower levels of anxiety among Chinese medical students in the postepidemic era. Similar findings were reported by Liu et al. [74], Rogowska et al. [66] found that while students' perceptions of stress showed a downward trend, anxiety levels showed a downward and then upward trend, which may be related to the continued decline in life satisfaction due to the epidemic. Duan et al. [75] also reported that insomnia and depression were common among Wuhan college students in the postepidemic era. They believe this may be due to the fear of a resurgence of the epidemic and the inconvenience caused by COVID-19. In addition, Michaeli et al. [56] argued that mental health symptoms may persist even after the pandemic is completely over and students return to their normal routines. Thus, compared to the early stages of the epidemic, students’ mental health may only partially improve, rather than fully return to preepidemic levels. We still need to keep an eye on the mental health of medical students.

## 8. Career intention

In this COVID-19 outbreak, the career intentions of medical students have also been affected in various ways. Gong et al. [76] found that after the outbreak, more students were willing to choose a clinical medicine major. Continued acknowledgment of healthcare staff from health authorities [77] and experience as volunteers during COVID-19 [78] might be the reasons why more medical students firm up their career intentions. 

Only a small proportion of medical students consider that their career intention will be influenced by COVID-19 [79]. One reason for this is that they were removed from the clinical setting during the pandemic. Removing students from the clinic may result in a loss of opportunities to explore the profession they are interested in, learn about various areas of medicine, build meaningful relationships with faculty in their intended specialty and receive guidance from professionals [80], [81]. Peng et al. [82] reported that psychological distress, lengthy medical education, heavy workloads, unsatisfied income, fierce competition, strained physician-patient relationships, declining social status of physicians, and worksite violence are notable reasons why medical students leave the medical profession. Apart from that, the mental damage caused by COVID-19, the perception that healthcare is a high-risk profession, and someone in the family having a medical background can all lead to students changing their career intentions [83]. 

## 9. Scientific research

COVID-19 has also had a major impact on scientific research. During COVID-19, many laboratory and clinical studies were suspended, and academic physicians were redeployed to the clinical setting to assist and treat COVID-19 patients [84]. Additionally, to prioritize COVID-19, research on COVID-19-related topics have been encouraged and funded and many non-COVID-19 projects has been suspended or canceled [85]. In addition, investment in COVID-19-related research increased during the pandemic [86], [87]. 

For the research that is still ongoing during the epidemic, the number of researchers entering the laboratory has been restricted, and laboratory meetings have been reduced or changed to virtual meetings to enhance social distancing [88]. Before the start of a study, researchers can explain research-related matters to participants and sign an electronic informed consent form through an online model [89]. In addition, in response to the temporary cessation of face-to-face visits, researchers provide remote access to participants via remote technology, use home testing or monitoring techniques, provide curbside or courier pickup and delivery of participant samples and study products, and update participants on study progress via phone, email, and e-health record portals [90]. Students can report research progress to their tutors through online methods such as ZOOM [91]. These measures not only ensure the normal conduct of research during the epidemic but also provide new methods for future research work.

## 10. International medical electives (IMEs)

IMEs are an important part of international exchange studies for medical students. IMEs are regarded as high-impact practices in clinical education [92]. Students learn in vastly different healthcare systems and cultures abroad, which will provide them with first-hand experience of global health [93]. However, due to the requirements of epidemic prevention work, some IMEs have been cancelled, which not only causes financial losses to students but, most importantly, deprives them of the opportunity to study in a new medical setting [94]. Egiz et al. [95] found that in Germany, the participation of students in medical electives abroad declined by 50% in 2020 versus 2019. The reasons mainly include lack of funding, travel restrictions, postponement of assistantship placements, and cancellation of electives [95], [96]. Meanwhile, the increased cost of electives, fewer places, greater competitiveness, and residency seat-to-applicant ratio are all likely to affect IMEs in the postepidemic era [97]. 

## 11. Limitations and perspectives

This review also has certain shortcomings. First, both the literature search and the data collection were carried out by only one author. Second, we can only summarize as fully as possible the impact of COVID-19 on medical students, and some aspects may still be lacking. Third, a summary of the opportunities presented by COVID-19 for medical students is lacking in this review. Furthermore, because the COVID-19 epidemic is not yet over, the potential long-term effects posed by COVID-19 on medical students still need to be explored.

## 12. Conclusion

The COVID-19 pandemic posed a great challenge to medical education. The online education model has helped to weather the educational crisis caused by COVID-19 but has many shortcomings. In the future, we should continue to improve the online education model and combine it with the offline education model more efficiently.

## Authors’ ORCIDs

- Wenwen Wang: 0000-0003-4008-0888
- Genpeng Li: 0000-0002-6848-5548
- Jianyong Lei: 0000-0001-7594-1671

## Author contributions

Wenwen Wang and Genpeng Li share first authorship. Wenwen Wang conducted literature retrieval and data collection. Wenwen Wang and Genpeng Li work together to summarize the information and write the first draft. These authors contributed equally to this work. Jianyong Lei took part in revising the article critically. Jianyong Lei substantial contributions to the conception and design. All authors approval for the version for publication; and agree to be accountable for all aspects of the work.

## Competing interests

The authors declare that they have no competing interests. 

## Supplementary Material

Strengths and weaknesses in online learning for medical students

Advantages and disadvantages of online exams

Factors affecting the mental health of medical students

Additional negative factors affecting the mental health of international students
