# Supplementary material for: The impact of COVID-19 on medical students
Source: GMS J Med Educ. 2024 Feb 15;41(1):Doc10. doi: 10.3205/zma001665 (PMC10946210; doi:10.3205/zma001665)
Supplement: Strengths and weaknesses in online learning for medical students [file JME-41-10-s-001.pdf]

Attachment 1: Strengths and weaknesses in online learning for medical students

Study	Year	Nation	Design	Strengths	Weaknesses
Chinelatto et al. [13]	2020	Brazil	Review	• More free time<br>• Useful for building time management skills	• Not applicable to clinical case discussion and practice<br>• Lack of accurate evaluation<br>• Heterogeneity of classroom quality<br>• Lack of human contact
Andersen et al. [14]	2022	America	Cross-sectional	• Increased learning flexibility<br>• More time with family and enjoy other interests	• Lack of in-person social interaction<br>• Unstable network connectivity<br>• Inability to access study materials on time<br>• No real clinical experience
Kaurani et al. [15]	2021	India	Web-based survey	• Abundant learning resources<br>• Facilitate a transformation of the learning process from teacher-led passive learning to student-centered active learning	• Overly fact-based teaching<br>• Not suitable for practical training
Kaur et al. [16]	2021	Punjab, India	Cross-sectional	• Comfortable study environment<br>• Flexibility<br>• Ability to continue learning during blocked periods<br>• Diverse learning content<br>• Access to digital expertise	• Family interruptions<br>• Lack of learning equipment<br>• Technical barriers<br>• Limited access<br>• Security problems<br>• Physical health problems, like visual fatigue, headaches
Bączek et al. [17]	2021	Polish	Cross-sectional	• Ability to stay at home<br>• Reduce the cost of accommodation and transportation<br>• Comfortable environment<br>• Sustainable access to online learning resources<br>• Permission to learn at your own rhythm<br>Ability to record sessions	• Absence of interaction with patients and teachers<br>• Technical issues with IT devices<br>• Lack of self-discipline<br>• Social isolation
Schlenz et al. [18]	2020	Germany	Cross-sectional	• More modern, fun, and accessible<br>• Less time effort<br>• Motivating learning<br>• Better knowledge delivery	• Cannot meet practical courses
Rajab et al. [19]	2020	Saudi Arabia	Cross-sectional	• Promote self-regulation and responsibility	• Communication and assessment issues
Mortagy et al. [20]	2022	Egypt	Cross-sectional	• More comfortable<br>• Reduced commute<br>• Permission to learn at one' s own rhythm<br>• More flexible and cost-effective	• Poor network connectivity<br>• Decreased communication<br>• Family disturbances

Study	Year	Nation	Design	• Strengths	• Weaknesses
Dost et al. [21]	2020	The United Kingdom	National cross-sectional	• No travel<br>• The ability to study at their own pace<br>• Flexibility<br>• More comfort<br>• Reduced costs<br>• Reduce anxiety<br>• Give students more time to prepare for clinical placement	• Internet connectivity issues<br>• Family distractions<br>• Increased equipment needs<br>• Additional study space required<br>• Difficulty concentrating and asking questions<br>• Weakening students' clinical capability and confidence<br>• Surging in choice<br>• Lack of communication
Baticulon et al. [22]	2021	Philippines	National cross-sectional	• No mentioned	• Increased equipment demand<br>• Limited access and network connectivity<br>• Lack of patient contact<br>• Excessive cognitive load<br>• Unlimited Internet access<br>• Family disruptions l<br>• Health problems<br>• Insufficient assessment<br>• Insufficient feedback from teachers
Saurabh et al. [23]	2021	India	Descriptive cross-sectional	• Study at home	• Limited student-instructor interaction<br>• Easily bored<br>• Lower classroom efficiency<br>• Not applicable to practical courses<br>• Inability to realize further study and comprehension<br>• Cause more stress
Stoller [24]	2021	America	SWOT analysis	• Enhance teamwork, participation, and communication	• Family distractions<br>• Loss of spontaneous interaction<br>• Increased demand for learning devices<br>• Lack of in-person interaction
Fan et al. [25]	2021	China	Review	• No mentioned	• Visual dysfunction
Tan et al. [27]	2022	China	Cross-sectional	• No need to adapt to a new environment<br>• Fewer social fears<br>• Provide tools to tackle language obstacles	• Disorders of daily life caused by jet lag<br>• Failure to feel the campus atmosphere of studying abroad
