# Supplementary material for: The impact of COVID-19 on medical students
Source: GMS J Med Educ. 2024 Feb 15;41(1):Doc10. doi: 10.3205/zma001665 (PMC10946210; doi:10.3205/zma001665)
Supplement: Advantages and disadvantages of online exams [file JME-41-10-s-002.pdf]

Attachment 2: Advantages and disadvantages of online exams

Study	Year	Nation	Design	Strength	Weakness
Gaur et al. [26]	2020	Bridgetown, Barbados	Review	• Attract students to learn on their own initiative	• Aggravate the problem of students' integrity in exams
Pace et al. [27]	2020	UK	Letter	• No mentioned	• Lead to the potential unfairness of the examination
Felthun et al. [28]	2021	Australia	Retrospective observational	• Can effectively perform most clinical skill assessments	• Unable to physical examination assessment
Alsoufi et al. [29]	2020	Libya	Cross-sectional	• Prevent delays in graduation and disruption of medical education	• Specific technical issues (e.g. need for specific cameras)<br>• The risk of test questions being leaked
Zagury-Orly et al. [30]	2021	Canada	Personal views	• Better assess students' thinking and reasoning skills<br>• Improve students' Internet application ability<br>• More conform to the requirements of the real clinical work environment<br>• Raise students' attention to evidence-based medicine<br>• Promote students to become self-directed learners	• Not suitable for assessing students' ability to understand and recall information

Study	Year	Nation	Design	Strength	Weakness
Mathieson et al. [31]	2020	UK	Letter	• Easier to track exam progress<br>• Reduce mistakes when filling out answer sheets<br>• Closer to work life<br>• More convenient and labor-saving<br>• Can timely test the learning situation<br>• Reduce anxiety	• More likely to cheat in exams
Jaap et al. [32]	2021	UK	Cross-sectional	• The exam atmosphere is more relaxed and conducive to reducing anxiety	• No exam atmosphere and difficulty concentrating<br>• Increase pre-test anxiety<br>• Require a quiet test environment<br>• Increase the inequity of exams
Atwa et al. [34]	2022	Bahrain	Mixed methods	• No mentioned	• Exam difficulty decreased<br>• Inaccurate assessment<br>• Potential cheating in the exam
Dhillon et al. [35]	2020	Canada	Observational	• No mentioned	• Potential academic integrity issues
Eckhardt et al. [36]	2021	UK	Cross-sectional	• Reduced exam fees (no additional accommodation and travel required)	• Potential Internet connection and equipment problems<br>• Lost the sense of special occasion<br>• Lack of face-to-face interaction and non-verbal communication
