# Supplementary material for: The impact of COVID-19 on medical students
Source: GMS J Med Educ. 2024 Feb 15;41(1):Doc10. doi: 10.3205/zma001665 (PMC10946210; doi:10.3205/zma001665)
Supplement: Factors affecting the mental health of medical students [file JME-41-10-s-003.pdf]

Attachment 3: Factors affecting the mental health of medical students

Author	Year	Nation	Mental health symptoms	Positive factors	Negative factors
Michaeli et al. [57]	2022	Germany	• Sleep problems<br>• Irritability<br>• Emotional instability<br>• Anhedonia<br>• Depression	• Social and emotional backing<br>• Exercise	• Social isolation<br>• Quarantine<br>• Increased daily screen time<br>• Poor network connection<br>• Insufficient hands-on activities
Li et al. [58]	2020	Canada	• Anxiety<br>• Stress	• Not mentioned	• Uncertainty about education and careers
Guldager et al. [59]	2021	Denmark	• Stress	• Living with parents	• Female<br>• Increased tasks<br>• Less than 21 years old<br>• Single<br>• Knew someone infected with COVID-19<br>• Afraid of being infected
Khan et al. [60]	2020	Bangladesh	• Anxiety<br>• Depression<br>• Stress	• Physical exercise<br>• Healthy Lifestyle	• Financial burden<br>• Fear of infection<br>• Inadequate food supply<br>• Insufficient information<br>• Overexposure to COVID-19 information
Alqahtani et al. [61]	2022	Saudi Arabia	• Anxiety<br>• Stress<br>• Fatigue<br>• Sleep problems	• Not mentioned	• Poor sleep quality

Attachment 3 to Wang W, Li G, Lei J. *The impact of COVID-19 on medical students*. GMS J Med Educ. 2024;41(1):Doc10. DOI: 10.3205/zma001665

Study	Year	Nation	Mental health symptoms	Positive factors	Negative factors
Tahir et al. [62]	2021	Pakistan	• Anxiety<br>• Depression	• Not mentioned	• Internet addiction<br>• Poor sleep quality
Cao et al. [63]	2020	China	• Anxiety	• Living in city<br>• Stable family income<br>• Living with parents<br>• Social backing	• Family or acquaintances infected with COVID-19<br>• Financial stress<br>• Disturbances to daily life<br>• Academic activities postponed
Alzahrani et al. [64]	2022	Saudi Arabia	• Anxiety<br>• Depression<br>• Stress	• Not mentioned	• Female<br>• Smoking<br>• Insufficient physical exercise<br>• Lower resilience<br>• Reduced immune status<br>• History of chronic and mental illnesses<br>• COVID-19-like symptoms
Xie et al. [68]	2020	China	• Anxiety<br>• Depression	• Provide adequate and accurate information about COVID-19<br>• Proper COVID-19-based knowledge training	• Overly concerned about the epidemic<br>• Informal information sources
Xiong et al. [69]	2021	China	• Anxiety<br>• Depression<br>• Stress	• Stress training<br>• Sense of control<br>• Reduction of perceived constraints	• Lack of ability to distinguish correct information

Study	Year	Nation	Mental health symptoms	Positive factors	Negative factors
Spatafora et al. [70]	2022	Germany	• Depression	• Trust in the healthcare system<br>• Provide student counseling and mental health care	• Fear of infection with COVID-19<br>• Female
Wu et al. [71]	2022	China	• Anxiety<br>• Depression	• Healthy diet<br>• Positive coping	• Unvaccinated<br>• Unhealthy diet
			• Sleep problems	• Completion of vaccinations	• Local family<br>• Negative coping<br>• Non-only child
Lyons et al. [72]	2020	Australia	• Psychological distress	• Resilience training<br>• Exercise<br>• Healthy diets<br>• Self-efficacy<br>• Rich interpersonal interaction<br>• Optimists<br>• Social support<br>• Emotional flexibility	• Negative effects of COVID-19 on daily learning and life<br>• Rising stress levels
Allen et al. [73]	2022	The United Kingdom	• Psychological distress<br>• Anxiety<br>• Loneliness	• Government's proactive response to the outbreak<br>• Trust in government<br>• Cultural beliefs<br>• Values<br>• Positivity	• Negative views and mindset<br>• Wrong guidance from the government<br>• Lack of PPE* and nucleic acid testing<br>• Low well-being<br>• Fake information

PPE: Personal protective equipment.
