# Supplementary material for: The impact of COVID-19 on medical students
Source: GMS J Med Educ. 2024 Feb 15;41(1):Doc10. doi: 10.3205/zma001665 (PMC10946210; doi:10.3205/zma001665)
Supplement: Additional negative factors affecting the mental health of international students [file JME-41-10-s-004.pdf]

Attachment 4: Additional negative factors affecting the mental health of international students

Author	Year	Nation	Mental health symptoms	Negative factors
Dodd et al. [78]	2021	Australia	• Anxiety<br>• Low well-being<br>• Low coherence	• Greater financial strain
Yuan et al. [79]	2021	China	• Anxiety<br>• Depression	• Language barriers<br>• Interpersonal difficulties<br>• Cultural differences<br>• Poor adaptability<br>• Increased frequency of late nights due to jet lag
Lin et al. [81]	2022	America	• Anxiety<br>• Depression	• Social and cultural differences<br>• Unfamiliarity with the new environment<br>• Jet lag problems lead to staying up late to study online<br>• Racial discrimination
Alam et al. [82]	2021	China	• Anxiety<br>• Depression<br>• Insomnia<br>• Stress<br>• Loneliness<br>• Fear	• Study in China for less than two years<br>• Further away from family
Sharma et al. [83]	2020	Australia	• Loneliness<br>• Anxiety<br>• Stress	• Jet lag affects sleep cycles<br>• Border restrictions may prevent access to family<br>• Accommodation problem<br>• Lack of contact with host country peers<br>• Heavier financial burden
